# Hydrogen-rich water inhibits glucose and α,β -dicarbonyl compound-induced reactive oxygen species production in the SHR.Cg-*Lepr*^*cp*^/NDmcr rat kidney

**DOI:** 10.1186/2045-9912-2-18

**Published:** 2012-07-09

**Authors:** Masanori Katakura, Michio Hashimoto, Yoko Tanabe, Osamu Shido

**Affiliations:** 1 Department of Environmental Physiology, Shimane University Faculty of Medicine, Izumo, Shimane, 693-8501, Japan

**Keywords:** Hydrogen-rich water, α,β-dicarbonyl compounds, Oxidative stress, Metabolic syndrome model, Advanced glycation end products

## Abstract

**Background:**

Reactive oxygen species (ROS) production induced by α,β-dicarbonyl compounds and advanced glycation end products causes renal dysfunction in patients with type 2 diabetes and metabolic syndrome. Hydrogen-rich water (HRW) increases the H_2_ level in blood and tissues, thus reducing oxidative stress in animals as well as humans. In this study, we investigated the effects of HRW on glucose- and α,β-dicarbonyl compound-induced ROS generation in vitro and in vivo.

**Methods:**

Kidney homogenates from Wistar rats were incubated in vitro with glucose and α,β-dicarbonyl compounds containing HRW, following which ROS levels were measured. In vivo animal models of metabolic syndrome, SHR.Cg-*Lepr*^*cp*^/NDmcr rats, were treated with HRW for 16 weeks, following which renal ROS production and plasma and renal α,β-dicarbonyl compound levels were measured by liquid chromatograph mass spectrometer.

**Results:**

HRW inhibited glucose- and α,β-dicarbonyl compound-induced ROS production in kidney homogenates from Wistar rats in vitro. Furthermore, SHR.Cg-*Lepr*^*cp*^/NDmcr rats treated with HRW showed a 34% decrease in ROS production. Moreover, their renal glyoxal, methylglyoxal, and 3-deoxyglucosone levels decreased by 81%, 77%, and 60%, respectively. Positive correlations were found between renal ROS levels and renal glyoxal (*r* = 0.659, *p* = 0.008) and methylglyoxal (*r* = 0.782, *p* = 0.001) levels.

**Conclusion:**

These results indicate that HRW inhibits the production of α,β-dicarbonyl compounds and ROS in the kidneys of SHR.Cg-*Lepr*^*cp*^/NDmcr rats. Therefore, it has therapeutic potential for renal dysfunction in patient with type 2 diabetes and metabolic syndrome.

## Background

Nonenzymatic glycation of proteins and the Maillard reaction result in the formation of advanced glycation end products (AGEs), which are associated with the pathogenesis of type 2 diabetes [1]. During the formation of AGEs, α,β-dicarbonyl compounds such as glyoxal, methylglyoxal, and 3-deoxyglucosone are produced as reactive intermediates [2]. Patients with type 2 diabetes have increased plasma levels of these compounds [3]. Moreover, AGEs [4,5], and α,β-dicarbonyl compounds [6] produce reactive oxygen species (ROS). Oxidative stress is of particular interest in the pathogenesis of diabetic nephropathy [7], the latter being the leading cause of end-stage renal disease.

Molecular hydrogen (H_2_), a potent free radical scavenger, selectively reduces the levels of the hydroxyl radical and peroxynitrite, which is the most cytotoxic ROS [8]. Consumption of water saturated with H_2_ [H_2_-rich water (HRW)] increases the blood H_2_ level and reduces renal oxidative stress in mice with cisplatin-induced nephrotoxicity [9], rats with chronic kidney disease [10], and rats with chronic allograft nephropathy [11].

We previously demonstrated that HRW confers considerable benefits against renal abnormalities in SHR.Cg-*Lepr*^*cp*^/NDmcr (SHRcp) rats, which are metabolic syndrome models, at least by preventing glomerulosclerosis and ameliorating creatinine clearance [12]. Furthermore, a sufficient supply of HRW may prevent or delay the development and progression of type 2 diabetes [13] and metabolic syndrome [14] by protecting against oxidative stress. However, the exact mechanisms of the beneficial effects of HRW on diabetic nephropathy remain unknown.

Given the potent free radical-scavenging activity of H_2_, we hypothesized that HRW may attenuate the production of α,β-dicarbonyl compounds as well as the production of ROS from AGEs and α,β-dicarbonyl compounds. In this study, we investigated whether HRW could inhibit glucose- and α,β-dicarbonyl compound-induced ROS production in vitro and in vivo.

## Methods

### Animals

Ten-week-old male Wistar rats (Clea Japan, Inc., Japan) were used for the in vitro experiments. Five-week-old male SHRcp rats (Disease Model Cooperative Research Association, Japan) were randomly divided into 2 groups: the HRW-treated group (n = 12), which received oral HRW, and the control group (n = 12), which received distilled water. Both groups were treated for 16 weeks as described previously [12]. All rats were housed under controlled temperature (23 ± 2°C)- and humidity (50% ± 10%)- with a 12-h light–dark cycle. The Wistar rats were fed normal rat chow (CE-2, Oriental Yeast Co., Ltd, Japan) while the SHRcp rats were fed Quick Fat (Clea Japan, Inc.) with ad libitum access to sterile-water or HRW ( Additional file 1: Table S1).

All rats were anesthetized with intraperitoneal sodium pentobarbital (65 mg/kg), following which their kidneys were removed, immediately frozen in liquid nitrogen and stored at −30°C until further use. The kidneys were homogenized with phosphate buffer (pH, 7.4) in a Teflon homogenizer. The homogenates were immediately frozen in liquid nitrogen and stored at −30°C until use. All animal experiments were conducted in accordance with the procedures outlined in the Guidelines for Animal Experimentation of Shimane University, compiled from the Guidelines for Animal Experimentation of the Japanese Association for Laboratory Animal Science.

### Generation of HRW

Nakao et al. have described the production and characterization of HRW [14]. HRW was prepared by dipping a plastic-shelled product (stick) comprising metallic magnesium (99.9% pure) and natural stones (Doctor SUISOSUI®; Friendear Inc., Japan) into distilled water. HRW was freshly prepared every other day in a 200-mL bottle containing the stick, and the H_2_ concentration was maintained between 0.3 and 0.4 ppm during the experiment.

### In vitro experimentation

#### Fenton reaction

Kidney homogenates were incubated with or without HRW in PBS containing 2 mM FeSO_4_ and 5 mM H_2_O_2_ for 60 min at 37°C. After the reaction, one aliquot was used for lipid peroxide (LPO) measurement and a second aliquot was added to ice-cold PBS for ROS measurement.

#### Treatment with glucose and α,β-dicarbonyl compounds

Kidney homogenates were incubated with or without HRW in PBS containing glucose (1, 10, or 100 mM; Wako Pure Chemical Industries, Japan), glyoxal (2, 20, or 200 μM; Nacalai Tesque, Inc., Japan), methyglyoxal (2, 20, or 200 μM; Sigma-Aldrich, USA), or 3-deoxyglucosone (2, 20, or 200 μM, Wako Pure Chemical Industries) for 60 min at 37°C. Ice-cold PBS was then added and ROS levels were measured.

### LPO measurement

LPO concentrations were measured using the thiobarbituric acid reactive substance assay as described previously [15], and they were expressed as moles of malondialdehyde. Malondialdehyde levels were calculated relative to a standard preparation of 1,1,3,3-tetraethoxypropane.

### ROS measurement

ROS levels were measured as previously described [16]. In brief, kidney samples were centrifuged at 12,500 g for 10 min at 4°C. The pellets were resuspended in PBS and sonicated for 3 min in ice-cold water. The substrate 2′,7′-dichlorofluorescin diacetate (Sigma-Aldrich) was mixed with the resultant samples, and fluorescence was monitored every 10 min for 60 min in the dark at 37°C. Fluorescence was measured with a DTX 880 multimode detector (Beckman Coulter, Inc., USA) using excitation and emission filters at 488 and 543 nm, respectively. 2′,7′-Dichlorofluorescein (Sigma-Aldrich) was used as the standard. Data were expressed as dichlorofluorescein production per minute per milligram of protein. Protein concentration was determined by using the Lowry method.

### Measurement of α,β-dicarbonyl compounds

The levels of α,β-dicarbonyl compounds in the kidneys and plasma of the SHRcp rats were measured as described with a slight modification [3]. In brief, glyoxal, methylglyoxal, and 3-deoxyglucosone solutions in 10 mM phosphate buffer (pH, 7.4) with 0.005% 3,4-hexanedione (Tokyo Kasei Organic Chemicals, Japan) as internal standard were incubated overnight with 0.01% 2,3-diaminonaphthalene (Tokyo Kasei Organic Chemicals) at 4°C. The reaction mixtures were extracted with ethyl acetate, and the organic layers were dried under nitrogen gas. The dried extracts were reconstituted with methanol and injected into an liquid chromatograph mass spectrometer (LC-MS/MS) system. HPLC was combined with ESI-MS in a TSQ Quantum mass spectrometer (Thermo Fisher Scientific K.K., Japan). HPLC was conducted in a Luna 5u C18(2) 100 Å LC column (150 × 2.0 mm, Phenomenex, USA) at 30°C. Samples were eluted with a mobile phase composed of acetonitrile methanol (4:1, v/v) and water acetic acid (100:0.1, v/v) in a 10:90 ratio for 5 min, ramped up to a 100:0 ratio after 25 min, and held for 10 min at a flow rate of 0.1 mL/min. MS/MS was conducted in positive ion mode, and 2,3-diaminonaphthalene derivatives of methylglyoxal (m/z 195 > 127), glyoxal (m/z 181 > 127), 3-deoxyglucosone (m/z 285 > 221), and 3,4-hexanedione (m/z 237 > 169) were detected and quantified by selected reaction monitoring.

### Statistical analysis

Results are expressed as means ± SEM. Data were analyzed by Dunnett’s multiple comparison test and Student’s *t*-test. Differences between groups were considered significant at *p*-values less than 0.05. All statistical analyses were performed with PASW Statistics 18.0 (IBM-SPSS, Inc., USA).

## Results

### Fenton reaction-induced ROS and LPO production

**Figure 1:**
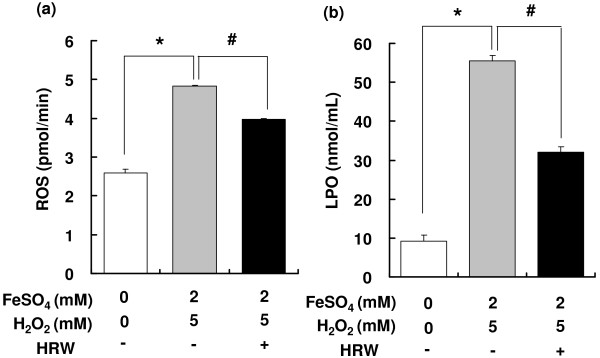
**Effects of HRW on Fenton reaction-induced ROS production.** Kidney homogenates from Wistar rats were incubated with (gray bars) or without (white bars) 2 mM FeSO_4_ and 5 mM H_2_O_2_ or HRW with 2 mM FeSO_4_ and 5 mM H_2_O_2_ (black bar), following which ROS (**a**) and LPO (**b**) levels were measured. Values represent means ± SEM (n = 3–6/group) ^*,#^*p* < 0.05 versus controls.

ROS and LPO levels by 82% and 62%, respectively, in the kidney homogenates incubated with H_2_O_2_ and FeSO_4_; however, their production was inhibited by treatment with HRW (Figure 1). These results are consistent with those of a previous study in which H_2_ selectively reduced the levels of the hydroxyl radical [8] generated by the Fenton reaction.

### Glucose- and α,β-dicarbonyl compound-induced ROS production

**Figure 2:**
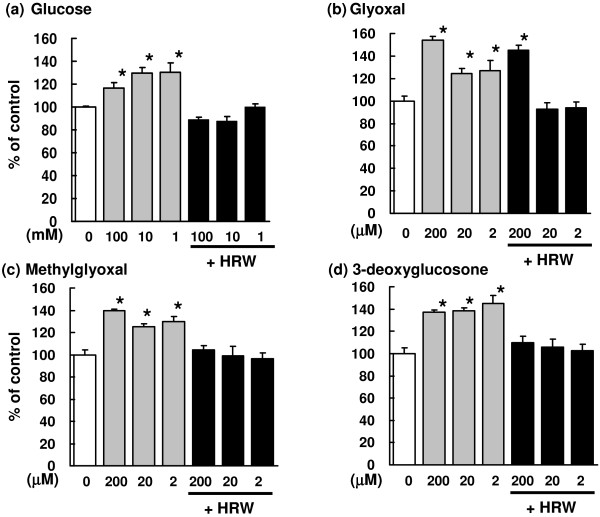
**Effects of HRW on glucose- and α,β-dicarbonyl compound-induced ROS production.** Kidney homogenates from Wistar rats were incubated with (**a**) glucose (1, 10, or 100 mM), (**b**) glyoxal (2, 20, or 200 μM), (**c**) methylglyoxal (2, 20, or 200 μM), or (**d**) 3-deoxyglucosone (2, 20, or 200 μM). Then, ROS levels were measured. Values represent means ± SEM (n = 3–6/group), ^*^p < 0.05 versus controls.

ROS levels were significantly increased after incubation with glucose or α,β-dicarbonyl compounds; however, their production was inhibited by treatment with HRW (Figure 2). These results indicate that HRW inhibited ROS production induced by glucose and α,β-dicarbonyl compounds.

### Renal ROS levels

**Figure 3:**
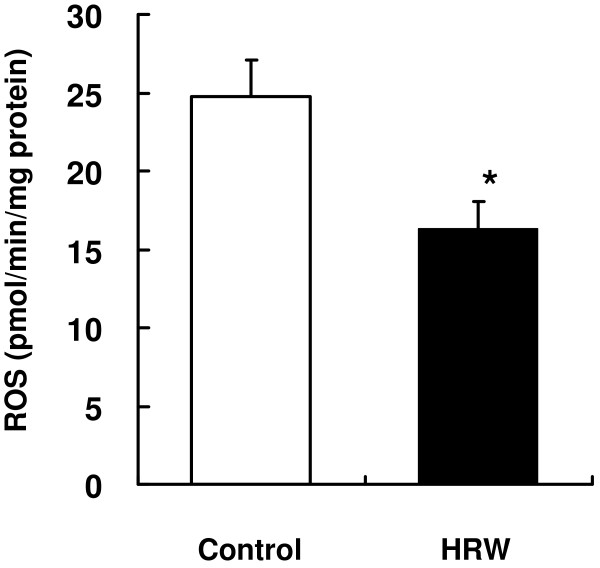
**Effect of HRW on renal ROS production in SHR.Cg- *Lepr* **^** *cp* **^**/NDmcr rats.** Values represent means ± SEM (n = 12/group), ^*^p < 0.05 versus controls.

As a result of the in vitro findings (Figures 1 and 2), renal ROS levels were measured in the SHRcp rats after treatment with HRW for 16 weeks. The renal ROS levels were significantly decreased by 34% in these treated animals compared with the controls (Figure 3), indicating that HRW inhibited ROS production in vivo.

### Plasma and renal α,β-dicarbonyl compound levels

**Figure 4:**
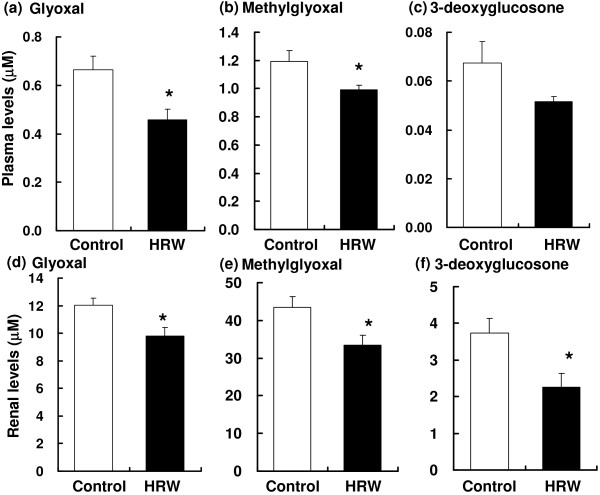
**Effects of HRW on plasma (a–c) and renal (d–f) glyoxal (a, d), methylglyoxal (b, e), and 3-deoxyglucosone (c, f) levels in SHR.Cg- *Lepr* **^** *cp* **^**/NDmcr rats.** Values represent means ± SEM (n = 12/group), ^*^p < 0.05 versus controls.

Glyoxal and methylglyoxal levels, but not 3-deoxyglucosone levels, were significantly decreased in the plasma of HRW-treated SHRcp rats compared with their control counterparts (Figure 4a–c). Furthermore, the renal levels of glyoxal, methylglyoxal, and 3-deoxyglucosone decreased by 81%, 77% and 60%, respectively, in the HRW-treated group (Figure 4d–f). Positive correlations were found between renal ROS levels and renal glyoxal (*r* = 0.659, *p* = 0.008) and methylglyoxal (*r* = 0.782, *p* = 0.001) levels; however, 3-deoxyglucosone levels (*r* = 0.202, *p* = 0.470) were comparable. These results indicated that HRW inhibit the production of α,β-dicarbonyl compounds and ROS in the kidneys of the SHRcp rats.

## Discussion

The present study revealed that HRW prevented glucose- and α,β-dicarbonyl compound-induced ROS production in vitro (Figure 2) and in vivo (Figure 3). In the in vivo study, HRW decreased the levels of α,β-dicarbonyl compounds in the plasma and kidney (Figure 4).

α,β-Dicarbonyl compounds reportedly form from degradation of glucose in 200 mM PBS (pH, 7.4) [17]. These compounds can produce free radicals [7,18] such as the hydroxyl radical [19], peroxynitrate [20], and the acetyl radical [21]. H_2_ selectively decreases the levels of the hydroxyl radical and peroxynitrite [8]. Data from the present study showed that HRW inhibited ROS production induced by the Fenton reaction and α,β-dicarbonyl compounds. Taken together, these results suggest that HRW decreases hydroxyl radical and peroxynitrate production induced by α,β-dicarbonyl compounds.

α,β-Dicarbonyl compound- and glucose-induced oxidative stress is considered to be a cause of renal dysfunction in vivo. Methylglyoxal and glucose induce renal oxidative damage and podocyte apoptosis in Zucker diabetic fatty rats [22] and *db/dB* mice [23]. Patients with type 2 diabetes have increased plasma levels of these compounds [3]. In the present study, we investigated whether HRW could inhibit α,β-dicarbonyl compound-induced oxidative stress in vivo. SHRcp rats were chosen for the present study because they exhibit several metabolic disorders, such as hypertension, hyperglycemia, hyperinsulinemia, and hyperlipidemia [24]. Histologically, islet area expansion, fatty liver, and glomerulosis can be observed in these rats. Therefore, they are considered to be a suitable animal model for renal dysfunction with the metabolic syndrome. Renal ROS levels were significantly decreased in the HRW-treated SHRcp rats compared with the control group (Figure 3). Furthermore, glyoxal and methylglyoxal levels in plasma and glyoxal, methylglyoxal, and 3-deoxyglucosone levels in the kidney were significantly decreased in HRW-treated animals compared with the control group (Figure 4). These results indicate that HRW inhibits ROS production by inhibiting α,β-dicarbonyl compound production.

Renoprotection does not necessarily depend on blood pressure or glycemic control [25]. Caloric restriction [26] and treatment with angiotensin II receptor blocker [27], pioglitazone [28], or cobalt [29] protect against renal dysfunction without blood pressure or glycemic control in SHRcp rats. These reports indicate that renoprotection is associated with decreased AGE formation and oxidative stress, thus suggesting that they are potential therapeutic strategies for renal dysfunction in patients with type 2 diabetes and metabolic syndrome. We previously reported that HRW does not affect blood pressure or blood glucose levels but prevents glomerulosclerosis in SHRcp rats [12]. In the present study, we noted that HRW inhibited the production of α,β-dicarbonyl compounds and ROS in these rats, suggesting that HRW has a potential therapeutic application for patient with renal dysfunction.

There are other possible molecular mechanisms to prevent oxidative stress cause by HRW. Kawamura et al. [30] have reported that in lung allograft in rats, H_2_ induces heme oxygenase-1 expression, decreases proinflammatory cytokines and the proapoptotic protein Bax, and increases expression of antiapoptotic protein Bcl-2. H_2_ reduces the binding of several transcription factors such as AP1 and NFκB to the iNOS promoter via inhibition of signal transduction in macrophages [31]. These reports suggest that the molecular target for HRW not only inhibits ROS generation but also induces gene expression of antioxidative enzymes at the transcriptional level. Further studies are necessary to explore these effects.

It has been reported that consumption of HRW for 8 weeks increases superoxide dismutase levels by 39% and decreases thiobarbituric acid reactive substance levels by 43% in the urine of subjects with potential metabolic syndrome [14]. Consumption of HRW is also associated with a significant decrease in the urinary levels of 8-isoprostanes, which are endogenous lipid peroxidation products [13]. These results indicate that HRW may have antioxidant activity in humans. Further studies are necessary to confirm the effects of HRW on renal dysfunction associated with type 2 diabetes and metabolic syndrome in humans.

Aminoguanidine, a prototype AGE formation inhibitor, acts by scavenging α,β-dicarbonyl compounds. Aminoguanidine has been shown to inhibit the formation of AGEs and slow the progression of diabetic nephropathy in animal models [32,33]. It also significantly decreases proteinuria in treated subjects [34]. However, aminoguanidine is a nonspecific AGE inhibitor that also inhibits nitric oxide synthase [35] and causes DNA damage [36]. Aminoguanidine cannot be used for the treatment of diabetic nephropathy because of safety concerns, and additional clinical studies are required to address the safety and efficacy of other types of AGE inhibitors [37]. On the other hand, consumption of HRW had no adverse effects on hematological parameters and biometric parameters during an 8-week study period in humans [14], suggesting that HRW is safe.

## Conclusions

In conclusion, HRW inhibits renal ROS production induced by glucose and α,β-dicarbonyl compounds in vitro and renal ROS and α,β-dicarbonyl compound production in vivo. Therefore, it has therapeutic potential for the treatment of renal dysfunction in patients with type 2 diabetes and potential metabolic syndrome.

## Abbreviations

AGEs, advanced glycation end products; HRW, H2-rich water; H2, Molecular hydrogen; LPO, lipid peroxide; ROS, reactive oxygen species; SHRcp, SHR.Cg-Leprcp/NDmcr.

## Competing interests

The author(s) declare that they have no competing interests

## Authors’ contributions

MK participated in the design of the study and carried out the incubation studies, LC/MS analysis, and drafted the manuscript. YT performed the statistical analysis. OS participated in the sequence alignment. MH conceived of the study, and participated in its design and coordination and helped to draft the manuscript. All authors read and approved the final manuscript.

## Supplementary Material

Additional file 1 ** Table S1.**Composition of the MF diet and Quick Fat diet Click here for file
