# Supplementary material for: Hydrogen-rich water inhibits glucose and α,β -dicarbonyl compound-induced reactive oxygen species production in the SHR.Cg-Leprcp/NDmcr rat kidney
Source: Med Gas Res. 2012 Jul 9;2:18. doi: 10.1186/2045-9912-2-18 (PMC3444324; doi:10.1186/2045-9912-2-18)
Supplement: Additional file 1 — Table S1.Composition of the MF diet and Quick Fat diet [file 2045-9912-2-18-S1.docx]

**Supplemental Table 1 Composition of the MF diet and Quick Fat diet.**

Composition for all ingredients is given in grams per 100 g of diet.

Ingredient MF diet Quick Fat

Moisture (%) 7.9 7.3

Crude protein (%) 23.1 24.0

Crude fat (%) 5.1 14.6

Crude fiber (%) 2.8 2.7

Crude ash (%) 5.8 5.1

Energy (kcal) 359.0 415.1
